# Supplementary material for: Factors influencing the E-learning system usage during the COVID-19 pandemic in Vietnam
Source: PLoS One. 2022 Dec 1;17(12):e0278109. doi: 10.1371/journal.pone.0278109 (PMC9714881; doi:10.1371/journal.pone.0278109)
Supplement: Attachment — Submitted filename: Response to Reviewers.doc [file pone.0278109.s001.doc]

September 20^th^, 2022

Subject: Revision submission (PONE-D-22-15672)

Dear Prof. Ogachukwu Chinedum Okoye,

We would like to send the revision of the article entitled "*Factors influencing the E-learning system usage during the COVID019 pandemic in Vietnam*" for publication in *Journal of Plos One.* We would like to thank the reviewer(s) and editor(s) for the valuable comments and suggestion. All the issues concerned were well revised and addressed:

**Reviewers' comments:**

**Reviewer 1:**

1. The research uses basic statistics. The research focuses one unique department of one university.

***Author's response****: At the time of the study, my university only specialized in Pharmacy. Therefore, the study was carried out throughout the school with all pharmacy students.*

2. The research misses recent literature review.

***Author's response****: We have updated recent studies (line 69, line 246-247; line 277-280).*

3. The research discussion is well stablished.

***Authors' response****: Thank you for your positive remarks on our manuscript.*

4. The conclusions need further development

***Authors' response****: We have developed more conclusions. Valuable lesson learned from E-learning period (Covid-19) will help HUP develop their distance learning programs to meet the learners’ demand in some diversity situation (line 306-308)*

**Reviewer 2:**

5. Line 48: Please list software properly - e.g. Zoom (Zoom Video Communications, San Jone, CA, USA)

***Authors' response****: Thank you for your comment. We have revised the software listing: Google Meet (Google, Mountain View, CA, USA), Microsoft Team (Microsoft, Redmond, DC, USA), Zoom (Zoom Video Communications, San Jose, CA, USA) (line 48-49)*

6. Most points should be simply reported in Intro and then discussed in the Discusion section

***Authors' response****: Thank you for your comment. We have reviewed and edited.*

7. Line 63-65: “many studies have been conducted to evaluate the education of health students” would be more broadly described as “many studies have been conducted to evaluate the education of health students and medical specialties”. At this point you can cite a recent international multicenter research work, such as Vascular e-Learning During the COVID-19 Pandemic: The EL-COVID Survey, Ann Vasc Surg 2021; 77: 63–70, DOI: 10.1016/j.avsg.2021.08.001

***Authors' response****: Thank you for your detailed comment. We have edited and added citations (line 67 and line 69)*

8. Line 72: What does “a convenience sample” mean?

***Authors' response****: We have added in the research method more clearly how to select the sample. Students presenting at the practical session or the theoretical session were recruited to the offline survey. The online form was only applied to final year students who finished the theory or practice at school. When participating in the online survey, the students accessed the questionaire through the group on the online platform of their class. Students accessing and participating in answering the online questionaires would be aggregated to analysis*. *(line 82-87).*

9. How did you make sure that participants did not submit an online and an offline survey form?

***Authors' response****: We have added more information in the research method.* *For each class, we only did offline or online form. The offline survey was applied for students from the 2^nd^ year to 4^th^ year who participated in the practical session or the theoretical session. Researcher collected the questionnaire form in the field, so we would control their response. (Line 79-82)*

10. Were the questionnaires validated?

***Authors' response****: We have added more information in the research method how to validate the questionnaires. (Line 103-110)*

11. How did you validate the online submitted data? Were there any duplicates?

***Authors' response****: We have added more information in the research method.* *The personal information in the online questionaire including name, year of birth, class, phone number and email was used to check for duplicates (Line 86-88). In addition, the online data was collected from only final year students, so there were not any duplicates*.

12. Line 100-104: Do you think that the filtering of the offline forms by the academic staff could result into any bias?

***Authors' response****: The questionnaires were distributed and collected by the reseach team. Therefore, we think that no questionnaires were filtered by the academic staff. We have added this information in the research method (Line 81-82 and 119).*

13. I was left with the impression that classic teaching was halted due to COVID. You mention that offline forms were distributed in classes and laboratories. The is a conflict in these statements. Please explain further

***Authors' response****: Due to the impact of the COVID pandemic, the online learning was applied for theoretical session at HUP in a period of time. However, when the pandemic was controlled, many Vietnamese university including HUP allowed students to return to school to learn directly. Then, the practical sessions were applied. Our research was carried out when students studied the theory and practiced directly at HUP (line 74-76)*

14. Apart from email distribution of the forms, did you include an online form within the e-learning platform

***Authors' response****: The collection data was conducted when students of HUP learnt directly at the school (the online learning stopped). Therefore, we only conducted online surveys through submitting a google form on the general class group and could not attach on the e-learning platform. We have added this information in the discussion section (line 290-294)*

15. Line 127-128: The online participation was lower than the offline participation. Wouldn’t this be strange when we are evaluating e-learning?

***Authors' response****: As mentioned above, the collection data was conducted when students of HUP learnt directly at school and the online learning at HUP stopped. We only conducted online surveys through submitting a google form on the general class group and could not attached on the e-learning platform. Therefore, not every students in the class had access our online questionnaires. This may lead to the low online response rate. The direct survey form was preferred to increase the response rate (line 290-295).*

16. Please include any segment necessary as per the above mentioned points

***Authors' response****: We have added the necessary discussions pointed out by reviewers (line 74-76)*

17. Please keep a consistent reference style

***Authors' response****: We have reviewed and edited to ensure consistency in reference styles.*

Thank you very much for your time and consideration. We hope that you are interested in our work.

Your sincerely,

Xuan Thang, Do

On behalf of all authors
