# Supplementary material for: Factors influencing the E-learning system usage during the COVID-19 pandemic in Vietnam
Source: PLoS One. 2022 Dec 1;17(12):e0278109. doi: 10.1371/journal.pone.0278109 (PMC9714881; doi:10.1371/journal.pone.0278109)
Supplement: Attachment — Submitted filename: Response to Reviewers.doc [file pone.0278109.s002.doc]

November 9^th^, 2022

Subject: Revision submission (PONE-D-22-15672R2)

Dear Prof. Ogachukwu Chinedum Okoye,

We would like to send the revision of the article entitled "*Factors influencing the E-learning system usage during the COVID019 pandemic in Vietnam*" for publication in *Journal of Plos One.* We would like to thank the reviewer(s) and editor(s) for the careful and detailed comments.

**Reviewers' comments:**

Please include a copy of Table 5 which you refer to in your text on page 14.

***Author's response****: We are sorry. Actualy, We have 4 tables in the Journal article. It is table 4 (not table 5). We have fixed it (line 180).*

Thank you very much for your time and consideration. We hope that you are interested in our work.

Your sincerely,

Xuan Thang, Do

On behalf of all authors
